# The ubiquitin conjugating enzyme, TaU4 regulates wheat defence against the phytopathogen Zymoseptoria tritici

**DOI:** 10.1038/srep35683

**Published:** 2016-10-19

**Authors:** Linda Millyard, Jack Lee, Cunjin Zhang, Gary Yates, Ari Sadanandom

**Affiliations:** 1 Department of BioSciences, University of Durham, Durham, DH1 3LE, United Kingdom.

## Abstract

*Mycosphaerella graminicola* (*Zymoseptoria tritici* commonly known as Septoria), the causal agent of Septoria Leaf Blotch (STB), is considered one of the major threats to European wheat production. Previous studies have shown the importance of ubiquitination in plant defence against a multitude of pathogens. However the ubiquitination machinery in wheat is under studied, particularly E2 enzymes that have the ability to control the ubiquitination and thereby the fate of many different target proteins. In this study we identify an E2 enzyme, *Triticum aestivum* Ubiquitin conjugating enzyme 4 (TaU4) that functions in wheat defence against Septoria. We demonstrate TaU4 to be a bona fide E2 enzyme through an E2 charging assay. TaU4 localises in both the cytoplasm and nucleus, therefore potentially interacting with E3 ligases and substrate proteins in multiple compartments. Virus Induced Gene Silencing of TaU4 in wheat leaves resulted in delayed development of disease symptoms, reduced Septoria growth and reproduction. We conclude that TaU4 is a novel negative regulator of defence against Septoria.

*Triticum Aestivum* (bread wheat) is one of the major food sources in many parts of the world and has been cultivated for more than 9,000 years^1^. Wheat yield is under threat from a devastating foliar fungal pathogen, *Mycosphaerella graminicola* (also known as *Zymoseptoria tritici* and *Septoria tritici*), which causes Septoria leaf blotch (STB)^2^. There have been reports of up to 50% yield loss due to Septoria infection. It has now been classed as one of the top ten most important pathogens to study by the international community^3^. Septoria is a hemibiotrophic fungus; it infects the leaf through the stomata and grows slowly as a biotroph for the first 12–14 days of infection. Then quickly, through an unknown mechanism, the fungus switches to a necrotrophic growth phase^4^, killing the leaf, causing loss of photosynthetic activity and therefore grain yield. During the biotrophic growth phase there are no infection symptoms, making it difficult to detect and treat before Septoria switches to necrotrophy, which kills the wheat leaves rapidly. Septoria then sporulates after around 28 days of infection and spreads through spores which can be carried by rain splashes and wind to other leaves on the plant^5^. In one wheat-growing season it has been predicated that Septoria can go through 6 life cycles^6^. Another major problem with preventing Septoria is its rapid evolution of resistance to new fungicides and resistant wheat varities^7^,^8^. Currently the most effective treatment for Septoria is to apply the fungicide before the initiation of infection. Very few fungicides are capable of stopping colonisation after initial infection^6^.

Due to plants sessile nature there is a need for rapid responses to stresses, such as pathogenic attack. One mechanism employed is posttranslational modification (PM) of proteins. Ubiquitination is a major peptide PM in plants. Attachment of ubiquitin to target proteins can cause them to become degraded by the proteasome, act as a signalling molecule or interact with different proteins amongst many other outcomes (reviewed in ref. 9). Components of the ubiquitination pathway represent a huge proportion of the plant proteome (>5% in *Arabidopsis thaliana*^10^); it is involved in the control of many aspects of plant life including (but not excluded to) plant defence.

The ubiquitin pathway is comprised of 3 enzymatic proteins (E1, E2 and E3), the ubiquitin molecule and a target protein. Firstly the E1 ubiquitin activating enzyme ‘charges’ ubiquitin using ATP. The C terminal glycine of the charged ubiquitin then becomes bound to the active site cysteine residue of the E1 enzyme via a thioester bound, forming a stable E1-ubiquitin intermediate.

The E1 then transfers the ubiquitin (via transesterification) onto the active site cysteine residue of the E2 ubiquitin conjugating enzyme (UBC). The E3 ubiquitin ligase enzyme confers specificity for the target protein by interacting with the target protein and the ubiquitin charged E2. The ubiquitin can then be transferred onto a lysine residue (via an isopeptide bond) of the target protein either directly or via an E3-ubiquitin intermediate depending on the class of E3 ligase (reviewed in ref. 9).

Cycles of ubiquitin conjugation can occur leading to chain formation. Varying chain lengths and linkages (through the 7 lysine residues of ubiquitin) can alter the target proteins fate (reviewed in ref. 9). For example when a chain of 4 or more ubiquitin molecules (bound through lysine 48 linkages^11^) attach to the target protein, this confers a signal to degrade the protein in the 26S proteasome^12^. This step may require a polybubiquitination enzyme otherwise known as an E4^13^.

The E3 ligase protein is believed to confer the specificity towards the target protein, however it has been discovered that the UBC enzyme can determine the target proteins fate by causing the different chain linkages and specify for initiation or elongation of a ubiquitin chain^14^. This may involve homo- or heterodimers of E2 enzymes being formed.

The number of enzymes in each of the 3 classes increases (E1 < E2 < E3) as the steps of the pathway progress, this is thought to allow increasing specificity for the target proteins leading to a larger number of possible combinations of possible outcomes^15^,^16^.

Plant immunity can be broken down into 2 main pathways, pathogen triggered immunity (PTI) and effector triggered immunity (ETI).

PTI is often referred to as basal defence. It allows the plant to recognise a multitude of pathogen attacks through common molecules, such as bacterial flagellin^17^. These molecules are known as Pathogen Associated Molecular Patterns (PAMPs). After recognition of the PAMPs by the plants Pattern Recognition Receptors (PRRs) many defensive traits are activated, including Reactive Oxygen Species (ROS) production and gene expression changes (reviewed in refs 18 and 19).

ETI defence has evolved after the evolution of pathogen effector molecules (encoded by Avirulence genes), which work to disrupt the plants ability to respond to or even detect the pathogen^20^,^21^. To counteract this, plants have co-evolved Resistance (R) genes which encode proteins that specifically recognise the pathogens effector proteins in a gene-for-gene relationship. Once ETI is triggered the plant often initiates a hypersensitive response (HR), leading to programmed cell death around the point of infection and stopping the infection spread. ETI also triggers systemic immunity in the rest of the plant (reviewed in ref. 19).

Ubiquitination has been shown to be involved in both PTI and ETI. For example, within PTI, Stegmann, *et al*.^22^ have shown that the E3 enzyme AtPUB22 is involved in attenuating the PAMP responses through ubiquitination of Exo70B2, a component of the exocytic machinery potentially involved in recycling of the membrane bound PRR signaling proteins, and therefore proteasomal degradation of Exo70B2. Due to the high-energy cost of plant defence tight controls are deployed to ensure mounting of the defence response is only activated when a pathogen is present.

*Cladosporium fulvum* is a fungal pathogen of tomato (*Lycopersicon esculentum*). Certain strains of *C. fulvum* express the Avirulence (Avr) gene, Avr9. Upon infection this can be recognized in a gene-for-gene relationship by tomato plants that express the Cf-9 resistance gene thus triggering ETI. However ETI recognition and defence can be broken down by the silencing of the E3 ligase LeACRE276, leading to no HR and a lowered defence response^23^.

Within crop plants ubiquitination has also been shown to important for defence.

For instance, overexpression of rice OsWRKY45 transcription factor confers strong resistance to both rice blast fungus and bacterial leaf blight^24^,^25^. In the absence of a pathogen OsWRKY45 is constitutively sent to the 26S proteasome for degradation after being tagged with ubiquitin. Upon pathogen attack OsWRKY45 is no longer targeted for degradation by ubiquitination, allowing rapid upregulation of defence related genes^26^. However there are few studies of ubiquitination in crops and even fewer of specifically E2 enzymes and their roles^26^,^27^,^28^,^29^,^30^.

In a screen to identify components of the ubiquitin system involved in Septoria responses in wheat we isolated mRNA from leaf tissue that was infected with Septoria. We isolated a particular mRNA that was predicted to encode a putative E2 enzyme from wheat, from here on termed, TaU4 based on sequence homology with known E2s. An E2 charging assay confirmed that TaU4 is an E2 enzyme. YFP:TaU4 localization is seen throughout the cell when visualized through confocal laser scanning microscopy (CLSM). Knocking down TaU4 gene expression using Virus Induced Gene Silencing (VIGS) led to later disease symptoms and reduced Septoria sporulation. To our knowledge this is the first report of ubiquitination involved in wheat defence against Septoria and this information could provide new control strategies against STB in wheat.

## Results

### TaU4 is an E2 ubiquitin conjugating enzyme

The E2 ubiquitin conjugating enzyme family all contain a 150–200 amino acid region known as the ubiquitin conjugating catalytic fold (UBC fold). E1, E3 enzymes and ubiquitin all interact and bind within the UBC fold^31^. There are four classes of UBC enzymes, Class I is the UBC fold as described, Class II has an N terminal extension, Class III has a C terminal extension and Class IV has both an N terminal and C terminal extensions^32^,^33^. These extensions have been implicated in localisation of the E2 enzymes^34^.

**Figure 1:**
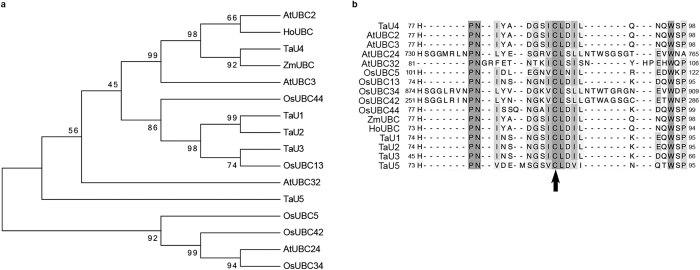
TaU4 The predicted protein sequence of TaU4 contains amino acid signatures reminiscent of ubiquitin E2 enzymes. Sixteen E2 enzymes were used for the alignment and phylogenetic tree construction, 4 from Arabidopsis (AtUBC2, AtUBC3, AtUBC24 and AtUBC32), 5 from rice (OsUBC5, OsUBC13, OsUBC34, OsUBC42 and OsUBC44), 1 from maize (ZmUBC), 1 from *Hyacinthus oreientalis* (HoUBC) and 5 from wheat (TaU1, TaU2, TaU3, TaU4 and TaU5). (**a**) Phylogenetic tree generated from aligning protein sequences of TaU4 against other bona fide plant UBC enzymes. The phylogenetic tree was constructed using Mega6 Molecular Phylogenetic analysis by Maximum Likelihood method with 1000 bootstrap replicates. (**b**) Multiple sequence alignments of TaU4 against the bona fide plant UBC enzymes. The arrow highlights the active site cysteine present in all the UBC enzymes. The alignment was performed using Webprank with the colouring representing sequence similarities between the UBC enzymes.

Following on from work previously described by Lee *et al*. (2015), an E2 enzyme (TaU4) was identified from a screen as being an ubiquitin system gene that has a potential role in Septoria infection. TaU4 is a small protein of 17.4 kDa (152aa), it is a member of the Class I UBC enzymes as it contains only the UBC fold^32^,^33^. A phylogenetic tree based on alignments of the amino acid sequence of TaU4 to 14 other plant UBC enzymes was generated (Fig. 1a). UBC enzymes were selected that represent each different class. TaU4 is most closely related to a maize UBC (ZmUBC), sharing 97% homology, whose function has yet to be identified. It is also closely related to *Arabidopsis thaliana* AtUBC2 (93% homology) and *Hyacinthus orientalis* HoUBC (91%), both of which have roles in flowering, with AtUBC2 controlling flowering time through the monoubiquitination of histones 2B^35^.

Within the UBC fold there is an active site cysteine residue, which binds to ubiquitin to form an UBC-ubiquitin intermediate^26^. TaU4′s active site cysteine was predicted to be at cysteine 88 based on alignments to the other plant UBCs. It is highlighted with an arrow in Fig. 1b. The shading in Fig. 1b also shows high conservation surrounding the active site. These amino acids function together in stabilising the fold surrounding the active site and facilitating ubiquitin binding to the cysteine residue^36^,^37^,^38^.

### TaU4 is a functional E2 enzyme

Since TaU4 has a similar protein sequence to other putative UBC enzymes a ubiquitin charging assay was performed to prove its E2 ability by binding ubiquitin to the active site cysteine through a thioester bond^15^,^39^. Ubiquitination involves a cascade of enzymes, for the ubiquitin-E2 intermediate to form it is essential to have an E1 enzyme, ubiquitin and ATP. The thioester bond is readily reduced, allowing easy transfer to the target protein either directly or via an ubiquitin-E3 intermediate^9^. With this knowledge the following controls were employed to prove the UBC function of TaU4; samples lacking E1 enzyme, ubiquitin, ATP and the addition of the reducing agent DTT. A well-studied human E2 enzyme (UbcH5b^40^) was used as a positive control. The commercially available E1 enzyme and UbcH5b are both His tagged proteins. GST:TaU4 and His:ubiquitin were both expressed in BL21 *E. coli* for 3 hours at 37 °C before affinity purification (Supplemental Figs 3 and 4).

**Figure 2:**
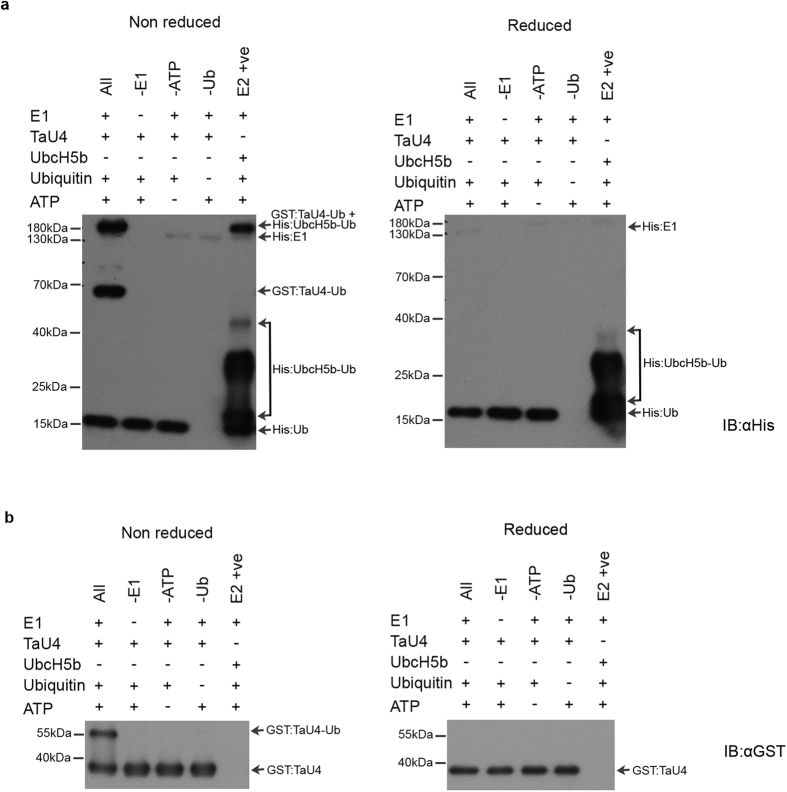
Thioester charging assays demonstrate that TaU4 is a bona fide ubiquitin E2 enzyme. (**a**) A western blot of E2 charging assay to show the binding of ubiquitin to TaU4. E1 enzyme, TaU4, ubiquitin and ATP were added together (All lane), followed by controls in which essential elements in the pathway to thioester formation on TaU4 were removed. For the positive control the E2 enzyme UbcH5b was used. The reducing agent DTT was added to the reduced samples (LHS) before running on a SDS-PAGE gel. Anti-His antibody was used to visualise the His tagged ubiquitin. Arrows indicate E1, E2 and His tagged Ub. (**b**) A western blot of E2 charging assay to show TaU4 becoming ubiquitinated on the active site cysteine (indicated by arrows). The same assay as in a probed with anti-GST to visualise GST:TaU4.

Anti-His antibody was used to visualise His:ubiquitin, whilst also allowing the E1 enzyme and UbcH5b to be detected due to the differences in molecular weights, (Fig. 2a) and anti-GST antibody (Fig. 2b) was used to visualise GST:TaU4.

Figure 2 shows ubiquitin binding to TaU4, this can be seen as an increase in the molecular weight of GST-TaU4. This increase is only seen in the lane containing all the essential components for ubiquitin binding. The thioester bond between TaU4 and ubiquitin was reduced (as can be seen by the lack of higher molecular weight bands in the reduced assays (Fig. 2a) after addition of DTT indicating a bona fide thioester bond formation between the active site cysteine of TaU4 and the terminal glycine of ubiquitin.

The positive control (UbcH5b) appears to readily bind ubiquitin (E2 +ve lane). After the addition of DTT there is a significant reduction in the amount of ubiquitin conjugated UbcH5b but not all thioester bonds are reduced in the reaction time frame. Our data demonstrates that TaU4 has the ability to bind ubiquitin and that it displays all the biochemical characteristics of a typical E2.

### *TaU4* is downregulated in Septoria infected wheat leaves during symptomless to symptom phase

Preliminary data indicated that there was a change in the expression of *TaU4* transcript levels in wheat seedlings 2 weeks after infection with Septoria. By investigating this further potential modes of TaU4 action can be concluded based on comparing expression changes with Septoria infection timings. To do this samples were collected from healthy and Septoria infected wheat leaves (4 week old seedlings) every 2 days over the whole infection period of 28 days. RNA was extracted from these samples and cDNA synthesised for qRT-PCR. *TaU4* gene specific primers, RT_TaU4A (Supplemental Table 1) were used to measure *TaU4* mRNA expression levels over the time course with primers specific for wheat *18S* gene was used as an endogenous housekeeping gene to normalisation of expression pattern.

**Figure 3:**
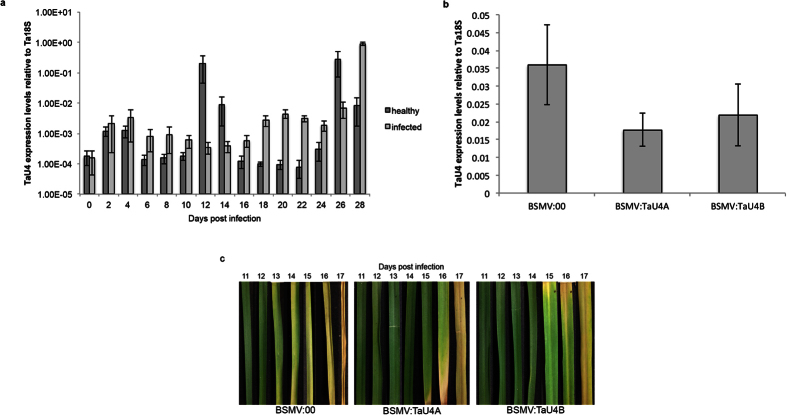
*TaU4* gene silencing in wheat affects STB symptom development. (**a**) Real-Time Polymerase Chain Reaction (RT-PCR) of *TaU4* expression in healthy and Septoria infected wheat leaves over 28 days of infection. *18S* was used as a housekeeping gene reference. Error bars represent ± 1 S.E.M. (**b**) RT-PCR to measure the levels of TaU4 expression in wheat leaves of BSMV:00, *BSMV:TaU4A* and *BSMV:TaU4B* silenced plants using TaU4A primers. Error bars represent ± 1 S.E.M. (**c**) White light pictures of Septoria infected *BSMV:00*, *BSMV:TaU4A* and *BSMV:TaU4B* plants were taken every day between 11–17 days post infection over the Septoria switch from biotrophic to necrotrophic growth and therefore the appearance of infection symptoms.

In the healthy wheat leaves *TaU4* transcripts show an cyclical pattern of increased expression followed by an down regulation every 8–12 days (Fig. 3a) over the course of the 28 day period. A similar pattern of expression is seen in the Septoria infected leaf samples with no significant difference when compared to uninfected leaves. However at 12 and 14 days post infection whilst *TaU4* expression in the healthy leaves increases rapidly, expression in the Septoria infected leaves is significantly supressed (Fig. 3a). This difference in expression between the healthy and infected leaves coincides with switch in Septoria lifecycle from biotrophic to necrotrophic growth. Thereafter *TaU4* expression continues to cycle similarly in both the healthy and infected leaves. This data indicates that *TaU4* may play a specific role in the switch to necrotrophy during Septoria lifecycle.

### *TaU4* silencing causes later infection Septoria infection symptoms and reduced sporulation

Due to wheat’s large hexaploid genome isolating single gene knockouts are not currently possible. Therefore Virus Induced Gene Silencing (VIGS) was employed to silence *TaU4*. Barley Stripe Mosaic Virus (BSMV) has previously been modified to effectively silence many different wheat genes^41^,^42^, including one involved in defence against Septoria^43^. Two independent non overlapping silencing fragments were used (*BSMV:TaU4A* and *BSMV:TaU4B*) to allow for different silencing efficiencies and to control for any unpredicted off target silencing (Supplemental Fig. 1). To check for transcript silencing RNA was extracted from the wheat leaves of *BSMV:00*, *BSMV:TaU4B* and *BSMV:TaU4A* treated plants two weeks after the initiation of gene silencing. From this RNA cDNA was synthesised for qRT-PCR, again using RT_TaU4A primers to measure *TaU4* expression. The RT_TaU4A primers do not amplify within either of the silencing fragments (Supplemental Fig. 1). Figure 3b shows the levels of *TaU4* expression after silencing treatment. After silencing with *BSMV:TaU4A* and *BSMV:TaU4B*, *TaU4* expression levels are 51% and 39% lower than the *BSMV:00* control respectively, indicating that *BSMV:TaU4A* is more efficient at silencing *TaU4* than *BSMV:TaU4B*.

The fresh weight and length of *BSMV:00*, *BSMV:TaU4A* and *BSMV:TaU4B* silenced leaves were measured 3 weeks after silencing treatment to assess whether TaU4 silencing caused a change in early development. Although it appears the average leaf length is slightly longer in TaU4 silenced plants, there is no significant difference when compared to the BSMV:00 control plants. This is again observed in the fresh weight of the leaves, with no significant difference being present between the silenced plants and the control plants (Supplemental Fig. 2).

**Figure 4:**
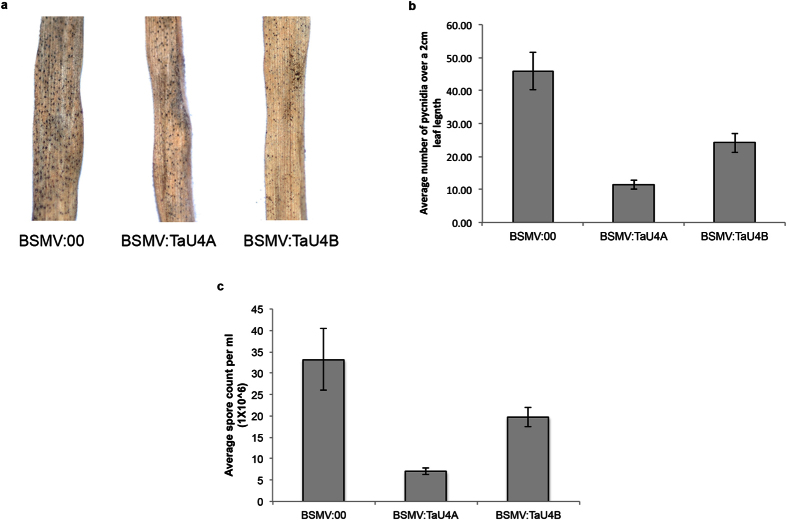
TaU4 gene silencing leads to reduced Septoria pycnidia formation in wheat. (**a**) White light pictures of pycnidia formation on *BSMV:00*, *BSMV:TaU4A* and *BSMV:TaU4B*. After 28 days of Septoria infection the infected wheat leaves were put into high humidity to induce the formation of Septoria pycnidia. (**b**) Average pycnidia counts from a 2 cm leaf length of Septoria infected *BSMV:00*, *BSMV:TaU4A* and *BSMV:TaU4B*. Mann Whitney U tests between *BSMV:00* and *BSMV:TaU4A* p value = 0.000016 (P < 0.05) and *BSMV:00* and *BSMV:TaU4B* p value = 0.002073 (P < 0.05). Error bars represent ± 1 S.E.M. (**c**) Average spore counts per ml (x10^6^) from Septoria infected *BSMV:00*, *BSMV:TaU4A* and *BSMV:TaU4B* leaves. Mann Whitney U tests between *BSMV:00* and *BSMV:TaU4A* p value = 0.000175 (p < 0.05) and *BSMV:00* and *BSMV:TaU4B* p value = 0.440313. Error bars represent ± 1 S.E.M.

14 days after silencing treatment the 3^rd^−5^th^ wheat leaves were subjected to Septoria infection (1 × 10^6^ spores per ml) and the symptom development was followed for 28 days (Fig. 3c) before pycnidia and spore counts to measure fungal development (Fig. 4b,c respectively). *TaU4* silenced plants displayed delayed Septoria infection symptoms when compared *BSMV:00* control (Fig. 3c). STB Symptoms start to develop after Septoria switches to its necrotrophic growth phase, this occurs at 13 days in *BSMV:00* control plants, however in *TaU4* silenced plants infection symptoms develop at 15 days with slower symptom progression in *BSMV:TaU4A* silenced plants in comparison to *BSMV:TaU4B*.

After 28 days of infection the infected leaves are put into high humidity to induce pycnidia formation (Fig. 4a) and therefore Septoria spore production. The pycnidia production was measured over 2 cm section of infected leaf. Figure 4 indicates that in *TaU4* silenced leaves Septoria pycnidia formation was significantly reduced, with 75% reduction in pycnidia on *BSMV:TaU4A* and a 48% reduction on *BSMV:TaU4B* silenced plants compared to *BSMV:00* control (Fig. 4b). As expected from the reduced number of fruiting bodies there is also a significantly reduced number of spores produced in the *TaU4* silenced wheat leaves, Fig. 4c, with a 79% reduction on *BSMV:TaU4A* and a 41% reduction on *BSMV:TaU4B* silenced plants when compared to the *BSMV:00* control. *BSMV:TaU4A* is more efficient at silencing *TaU4* than *BSMV:TaU4B*. This correlates to the slightly delayed infection symptoms and lower pycnidia and spore count in the *BSMV:TaU4A* silenced plants when compared to *BSMV:TaU4B*.

### TaU4 is a small protein that can diffuse throughout the cell

**Figure 5:**
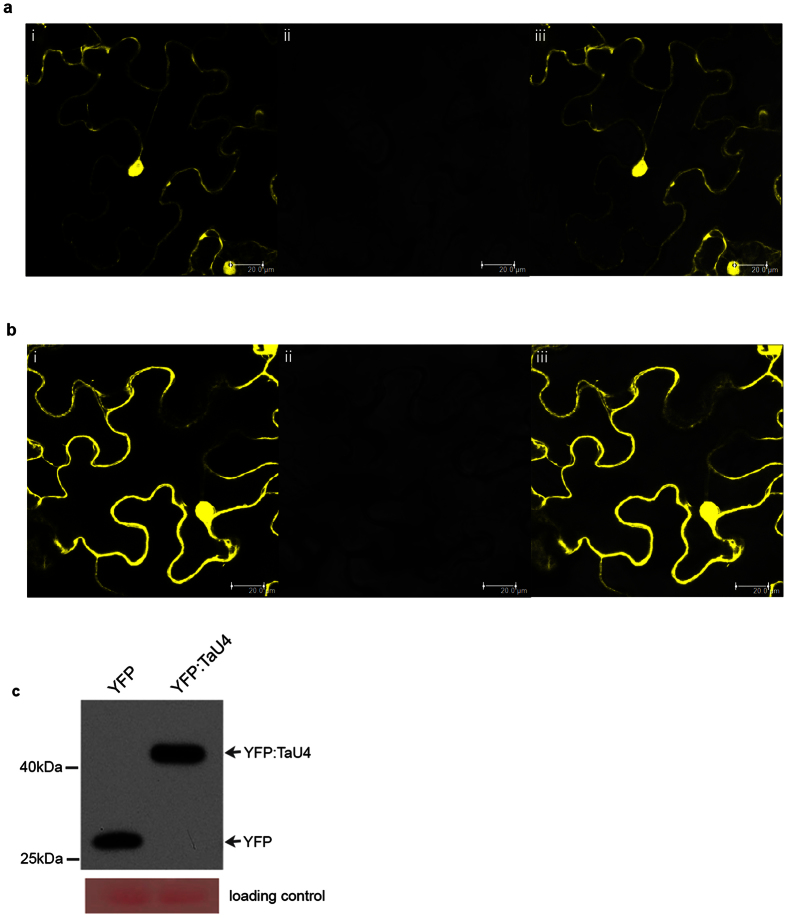
Cellular localisation of YFP:TaU4. YFP-TaU4 localises to the cytosol and nucleus in *N. benthanmiana* leaf cells. (**a**) YFP localisation throughout the cell. YFP (PearleryGate104) was transiently expressed in *N. benthanmiana* leaves and visualised using confocal laser scanning microscopy **i** YFP fluorescence detected at 560–700 nm. **ii** Brightfield image. **iii** Overlay of the previous two channels. (**b**) YFP:TaU4 fluorescence is detected throughout the cell, similar to YFP. (**c**) A western blot of YFP and YFP:TaU4 extracted from *N. benthanmiana* plants infiltrated with PearleryGate104 and PearleryGate104-TaU4 respectively. The blot was probed with anti-YFP to show the fusion of YFP:TaU4.

To ascertain the site of action within the cell of TaU4 was fused to an N-terminal Yellow Fluorescent Protein (YFP). The DNA construct was transiently expressed (under the 35S promoter) in *Nicotiana benthamiana* and the subcellular localisation of TaU4 was visualised with Confocal Laser Scanning Microscopy (CLSM). YFP control was localised to both the membrane and the nucleus (Fig. 5a) with TaU4 also observed to have a similar localisation pattern throughout the cell at both the cytoplasm and nucleus (Fig. 5b). YFP only and YFP:TaU4 fusion proteins were extracted from *N. benthamiana* for western blotting to ascertain that the fusion protein was correctly expressed. YFP:TaU4 recombinant fusion protein migrated at a molecular weight of 44 kDa whereas YFP was at 26 kDa. Figure 5c shows the difference in molecular weight between YFP and YFP:TaU4 fusion protein therefore demonstrating that the localisation visualised in Fig. 5b is due to YFP:TaU4 and not YFP only fragment from YFP:TaU4 degradation. Our data indicates that TaU4 is present throughout the cell and does not appear to accumulate in a specific subcellular compartment. YFP does not mask TaU4s localisation as the C-terminally tagged TaU4:YFP also localises throughout the cell (Supplemental Fig. 5).

Ubiquitin can be linked through any one of its 7 lysines to form polyubiquitin chains. These different linkages lead to different fates for the ubiquitinated target protein. For instance, a chain of more than 4 ubiquitin’s linked through lysine48 targets a protein for degradation in the 26S proteasome^10^. An example of K63 linkage mediated outcomes are the targeting of ubiquitinated proteins towards the lysosome for degradation^44^ and the involvement in stress responses in yeast^45^. The E2 enzymes are thought to determine which of the lysines the linkage occurs through, therefore determining the outcome for the target protein. By extracting the total protein from *BSMV:00*, *BSMV:TaU4A* and *BSMV:TaU4B* silenced wheat leaves the amounts of the 2 most studied polyubiquitin chain linkages (lysine48 and lysine63) could be assessed through western blotting using antibodies specific to poly-K48 or –K63 chains. It appears that patterns of global K48 or K63 polyubiquitin chain linkages are not significantly affected in the *TaU4* silenced plants (Supplemental Figures 6 and S7). Although in the K63 linkage polyubiquitin chains blots we did detect an increased intensity of a band migrating at approximately 60 kDa in the *TaU4* silenced lines. This data suggests that TaU4- E2 may affect K63 linked polyubiquitin chains on specific targets in wheat rather than global polyubiquitin chains. The identity of the target(s) may prove to be important in understanding the role of E2s in wheat –Septoria interaction but is beyond the scope of the current study.

## Discussion

TaU4 was predicated to be a UBC enzyme based on alignments to previously identified E2 enzymes from other plant species. TaU4 is a small ubiquitin conjugating enzyme at only 152 amino acids and is part of the Class I family of UBC enzymes. Class I UBC enzymes contain just the UBC fold, whereas class II and III have N and C terminal extensions respectively and class IV has both side extensions. These extensions are thought to contribute to the regulation and action of the E2 enzyme^46^,^47^. It has a very close homologue in maize (ZmUBC), sharing 97% amino acid identity, *Arabidopsis thaliana* (AtUBC2), 93% identity and 91% with *Hyacinthus orientalis* (HoUBC). All UBC enzymes have an active site cysteine that the ubiquitin binds to forming the intermediate ubiquitin-UBC conjugate. Based on alignments to the other UBCs (Fig. 1b) the active site for TaU4 was predicted to be cysteine 88^36^,^37^,^38^.

As TaU4s interacting E3 partners and target proteins are unknown the formation of an E2-ubiquitin intermediate was used to prove its UBC function. TaU4’s ability to form a TaU4-ubiquitin intermediate is shown in Fig. 2. As the bond between ubiquitin and TaU4 could be broken under reducing conditions (produced by adding DTT) this shows that the ubiquitin was bound through a thioester bond onto the suspected active site cysteine 88 (as in the ubiquitination pathway) and it is not being ubiquitinated through a peptide bond onto a lysine residue as with proteins being targeted for ubiquitination.

Previous studies have shown the importance of ubiquitination in defence against pathogens^22^,^23^,^26^,^27^,^28^,^48^,^49^, however most of the research is focused on E3 ligases and their targets. To our knowledge this is the first report of a wheat UBC enzyme involved in wheat defence against Septoria.

VIGS^41^,^42^ was employed to silence *TaU4*, with two independent silencing fragments (*BSMV:TaU4A* and *BSMV:TaU4B*) being used to ensure off target silencing wasn’t involved in the phenotypes observed. The silencing of *TaU4* does not completely repress transcript levels, with *BSMV:TaU4A* treatment leading to 51% silencing and *BSMV:TaU4B* causing 39% silencing (Fig. 3b). Even with this moderate silencing treatment there is still a distinct infection phenotype (Figs 3 and 4). Lower expression of *TaU4* causes delayed Septoria infection symptoms (Fig. 3c) and a decrease in the amount of pycnidia (Fig. 4a,b) and spores produced by the fungus (Fig. 4c). *BSMV:TaU4A* has a higher silencing efficiency, this can be correlated with the less Septoria sporulation and pycnidia when compared to *BSMV:TaU4B*.

Ubiquitination is not only important for plant defence responses; it is also implicated in plant growth. One well-defined growth process that is controlled by ubiquitination is its involvement with gibberellin hormone (GA). GA binds to the GA receptor, GID1. When GID1 binds to GA it undergoes a conformational change allowing it to interact with growth repressor DELLA. When GID1 is bound to DELLA this signals for a SCF^GID1^ E3 ligase complex to polyubiquitinate DELLA, leading to its degradation and upregulation of genes involved in growth.

*TaU4* undergoes oscillations in expression, with each lasting between 8–12 days (Fig. 3a). This expression pattern hints at a possible role within growth and development, such as those mentioned above. During the undetectable^50^,^51^ biotrophic phase of Septoria growth there is no difference in *TaU4* expression in infected leaves compared to healthy leaves. However as Septoria switches to necrotrophic growth and becomes detectable in the plant (1 dpi) *TaU4* expression is suppressed in the infected leaves. *TaU4* expression within the infected leaves then returns to the oscillation pattern seen in healthy leaves. A plausible explanation would be that after detection of a pathogen the plant needs to rapidly switch resources from growth to defence, we hypothesize that TaU4 is critical for this switch to defence. This is supported by our VIGs data (Figs 3 and 4) indicating that suppression of *TaU4* affects Septoria growth and development in wheat.

We postulate that *TaU4* is a positive regulator of growth therefore is downregulated upon the Septoria infection to upregulate defence responses. VIGS does not lead to a complete knock out in *TaU4* transcription. This lowering of expression can allow *TaU4* to still act within the cell but to a lesser degree, therefore potentially priming the silenced plants for the defence response. This leads to reduced Septoria growth (Fig. 4) when infecting *BSMV:TaU4A* and *BSMV:TaU4B* silenced plants in comparison to *BSMV:00* control.

TaU4 is localised throughout the nucleus and cytoplasm (Fig. 5b). It is a small protein of 17 kDa, (44 kDa with the YFP tag). Proteins of 17 kDa (and 44 kDa) are considered small enough to be able to passively diffuse through the nuclear membrane^52^,^53^,^54^, therefore it is unsurprisingly that YFP:TaU4 is localised throughout the cytoplasm and nucleus. Other E2 enzymes (UbcM2) have also been found throughout the cell, however UbcM2s localisation in not passive and is dependent upon its ubiquitination status^55^. TaU4′s localisation throughout the cell implies a wide range of interacting E3 ligases and target proteins.

Wheat populations in temperate regions are under threat due to the devastating foliar pathogen *Zymoseptoria tritici*. New resistant varieties are needed in combination with new fungicides to stay one step ahead of the rapidly evolving pathogen. The ubiquitin system is one potential target for crop breeders, with ubiquitin already implicated in plant defences across both model and crop species. We have shown that TaU4 UBC enzyme is involved in wheat defence and that downregulating its expression can have significant effect on STB disease development. Further work is needed to identify the downstream effects of silencing TaU4 and elucidate other components of the pathway.

## Methods

### Phylogenetic analysis

The online alignment program webPRANK^56^ was used to generate an amino acid alignment, which was then edited using the software Jalview^57^. The evolutionary history was inferred by using the Maximum Likelihood method based on the Jones *et al*. w/freq. model^58^. The bootstrap consensus tree inferred from 1000 replicates is taken to represent the evolutionary history of the taxa analyzed^59^. The initial tree for the heuristic search was obtained by applying the Neighbor-Joining method to a matrix of pairwise distances estimated using a JTT model. A discrete Gamma distribution was used to model evolutionary rate differences among sites (5 categories (+G, parameter = 5.5679)). The rate variation model allowed for some sites to be evolutionarily invariable ([+I], 4.3710% sites). The analysis involved 16 amino acid sequences. All positions containing gaps and missing data were eliminated. There were a total of 101 positions in the final dataset. Evolutionary analyses were conducted in MEGA6^60^.

### Plant material and growth conditions

*Triticum aestivum* cv. Avalon was grown in an environmentally controlled room at 24 °C in 16:8 hours light:dark cycles. Leaf material for RNA extraction was collected 8 hours into the light cycle. *Nicotiana benthanmania* was grown in an environmentally controlled room at 20 °C in 18:8 hours light:dark cycles.

### qRT-PCR

RNA was extracted using Zymoresearch RNA extraction kit and cDNA synthesis was performed using Superscript II (Invitrogen).

All quantitative real time PCR (qRT-PCR) was performed in a 20 μl reaction volume with SYBR green (Sigma) with 250 ng of cDNA. Wheat 18S gene (*Ta18S*) was used as an endogenous control. Three independent biological repeats and three independent technical repeats were performed for each experiment. Primers are listed in Supplemental Table 1.

### Pathotests

*Zymoseptoria tritici* isolate IPO323 was used for the pathology tests. Septoria spores were grown on agar plates supplemented with yeast extract, peptone and dextrose plates for 5 days at 18 °C. Spores were suspended in water with 0.1% Tween20 at a concentration of 1 × 10^6^ for the plant infection. 28-day-old wheat plants were then infected with the Septoria. After 28 days of infection the plants were then put into high humidity conditions (>80% humidity) to induce pcynidia and spore production. The pcynidia were counted over a 2 cm leaf length (3 biological repeats and 10 technical repeats). Replicates of 5 leaves were submerged in 10 ml of deionized water, the spores washed off and collected for counting on a haemocytometer and light microscope.

### Virus Induced Gene Silencing (VIGS)

Knock down studies were performed on *Tritium aestivum* using Virus Induced Gene Silencing (VIGS) technique^41^. An adapted Barley Stripe Mosaic Virus (BSMV) expressing a fragment of the target gene was used to knock down the expression in the wheat as previously described^42^. Silencing constructs were analyzed for their specificity and efficiency using siRNA finder si-fi (labtools.ipk-gatersleben.de/index.html). Two silencing fragments were employed due to the possible off target silencing and differing silencing efficiencies (*BSMV:TaU4A* and *BSMV:TaU4B*).

### Agrobacterium mediated transient assays and Confocal Microscopy

Transient expression of the Yellow Fluorescent Protein (YFP) gene, YFP:TaU4 and TaU4:YFP (pEARLEYGATE104 and pEARLEYGATE 101 respectively) was performed by infiltrating the constructs into 8 week old *Nicotiana benthamiana* leaves. After 3 days small sections of the infiltrated leaves were viewed under a x63 objective in a Confocal Laser Scanning Microscope (Leica SP5 CLSM, Berlin, Germany) to determine the sub cellular localization of each construct.

### E2 charging assay and protein purification

His:ubiquitin and GST:TaU4 were expressed in BL21 *E. coli* cells at 37 °C until the O.D. 600 reached 0.6 when 0.1 μM of IPTG was added. Cells were then harvested 2 hours after induction for purification (Supplemental Fig. 3). His:ubiquitin was purified using HisTrap HP columns (GE healthcare) and GST:TaU4 was purified using GSTrap4B columns (GE healthcare) (Supplemental Fig. 4). The His:E1 ubiquitin activating enzyme and the positive control E2 ubiquitin conjugating enzyme (His:UbcH5b) were purchased from Enzolife sciences.

The following was combined and incubated at 30 °C for 5 minutes. 1.25 μl of E1 enzyme (1.6 μg/μl), 1 μl E2 enzyme (TaU4/UbcH5b)(2 μg/μl), 2 μl 10X thioester buffer, 2 μl of ubiquitin (3.75 μg/μl) and made up with water up to 20 μl. 20 μl of 2x non-reducing loading buffer for SDS PAGE was added. For the reduced sample, 0.8 μl of DTT 1 M was added to 20 μl of the reaction mixture. The reduced samples were then boiled for 5 minutes at 98 °C before loading onto the gel. The remaining 20 μl (non reduced sample) was incubated at 30 °C for a further 15 minutes before loading onto a12% SDS PAGE gel. Technique modified from^39^.

The proteins were then visualized through western blotting method using anti-His (1:10,000 for 2 hour incubation) and anti-GST (1:5,000 for 2 hour incubation).

## Additional Information

**How to cite this article**: Millyard, L. *et al*. The ubiquitin conjugating enzyme, TaU4 regulates wheat defence against the phytopathogen Zymoseptoria tritici. *Sci. Rep.* **6**, 35683; doi: 10.1038/srep35683 (2016).

## Supplementary Material

Supplementary Information
