# Supplementary material for: The ubiquitin conjugating enzyme, TaU4 regulates wheat defence against the phytopathogen Zymoseptoria tritici
Source: Sci Rep. 2016 Oct 19;6:35683. doi: 10.1038/srep35683 (PMC5069635; doi:10.1038/srep35683)
Supplement: Supplementary Information [file srep35683-s1.pdf]

The ubiquitin conjugating enzyme, TaU4 regulates wheat defence against the phytopathogen Zymoseptoria tritici.

Linda Millyard[1], Jack Lee[1], Cunjin Zhang[1], Gary Yates[1] and Ari Sadanandom[1*].

[1] Biological and Biomedical Sciences, Durham University, Durham, DH1 3LE

[*] To whom correspondence should be addressed. Email:

ari.sadanandom@durham.ac.uk

TaU4

```
GATCCCCGACCCTCCCTTCCATGCCCAGGCCGACCTAAAGCCGTCCATTCCGTCGTCTCCAT
GCTCTAGGACAACGAGCACGCGGAGCCCACGTCTCCACCCCCACCGCCGGCCGCATCTCAG
AGAATTTCGAGGATGTCGACTCCTTCAAGGAAGAGGCTGATGAGGGACTTCAAGCGGCTG
ATGCAGGACCCTCCTGCGGGCATAAGCGGGGCGCCGCAGGACAACAACATAATGCTGTGG
AATGCTGTGATTTTTGGCCCTGACGATAGCCCGTGGGATGGAGGCACGTTTAAGCTGACTC
TCCAGTTTAATGAAGAATATCCTAATAAGCCACCAACAGTTCGGTTTATTTCTCGGATGTTT
CACCCTAACATTTATGCTGATGGAAGCATATGCTTAGATATTCTACAGAATCAGTGGAGCCC
AATATATGATGTAGCTGCTATACTTACATCTATCCAGTCGCTGCTGTGTGATCCTAACCCAA
ATTCGCCTGCTAACTCAGAAGCTGCCCGCATGTTCAGTGAGAACAAGCGAGAGTACAACCG
CAAAGTGCGGGAGATTGTTGAGCAGAGCTGGACGGCAGACTAATAAGTTGAGCTCACCAT
GTGTTATCATGCGGTTTCTGTACCAAAATGTTTGTAACTAAAATGACTGAACTCTGTCGTTA
CCACCTGAAACAGCAACTTGCTTTGTTGCATCGTTTGGCTGGACGGTTGGAGATTGCTCTG
TCATGGCCTGTGTTTACACTGTCTGTATCTGGAACGCAAACATTTTGTAACGTCTTGTGTGA
GTTTGTAATGTTGTATCATATCACATTGATTCTGAAAAAAAAAAAAAAACGA
```

Supplemental Figure 1. TaU4 mRNA sequence to indicate position of silencing fragments and qRT-PCR primer amplification regions. ATG and TAA stop codons are highlighted in black. Highlighted in grey are the silencing fragments (with *BSMV:TaU4A* within the 5’ UTR and N terminus and *BSMV:TaU4B* at the C terminal region). The qRT-PCR amplification regions are underlined.

a.

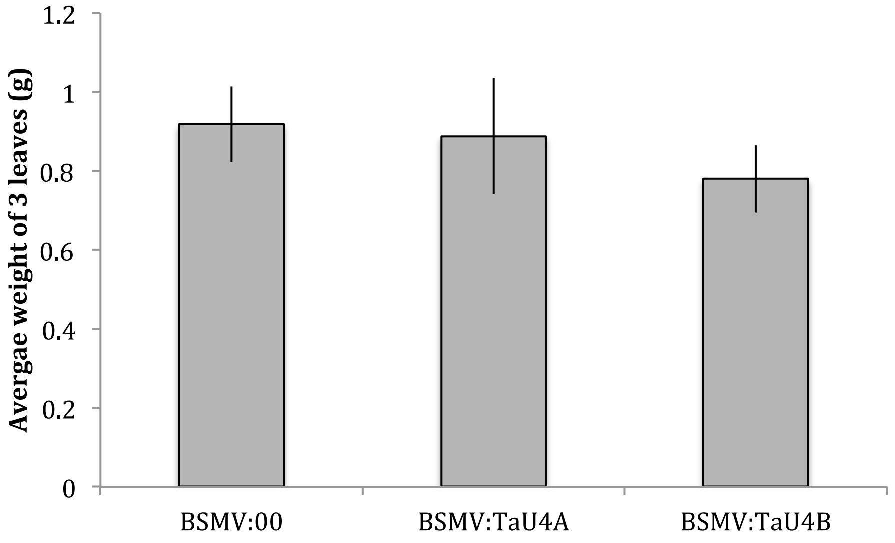

b.

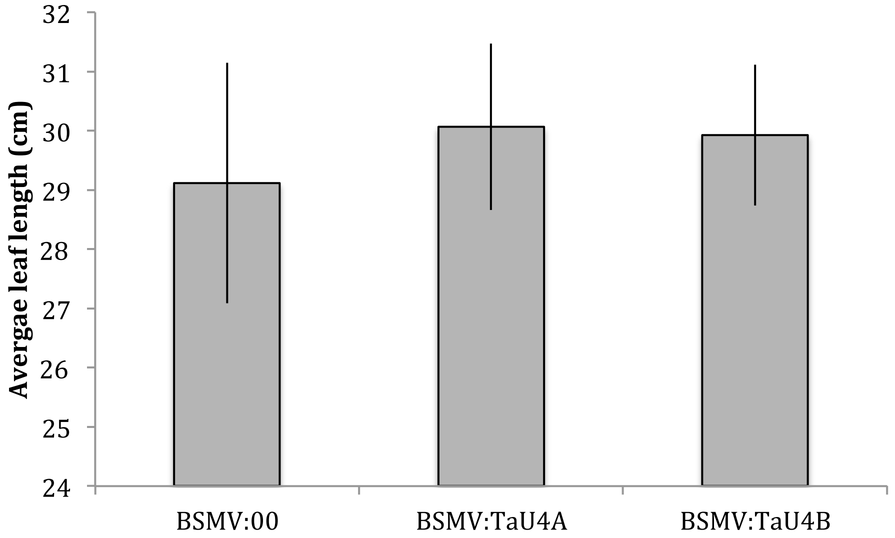

Supplemental figure 2. Fresh weight (a) and the length (b) of *BSMV:00*, *BSMV:TaU4A* and *BSMV:TaU4B* were measured 3 weeks post silencing treatment. 15 leaves from 5 different plants were measured (3-5 leaves). A Mann-Whitney U test was performed and no significant differences were found between BSMV:00 and TaU4 silenced plants.

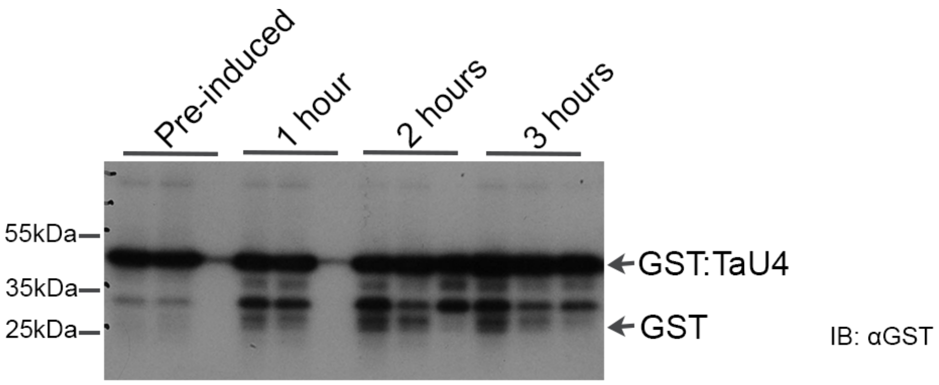

Supplemental figure 3. Western blot to show expression of GST:TaU4. The pre-induced is shown, after which 1mM of IPTG was added and samples collected every hour for 3 hours. Each time point shows the total, soluble and insoluble protein fractions respectively. GST:TaU4 is 41kDa and GST is 26kDa. An anti:GST antibody was used to visualise the proteins through western blotting.

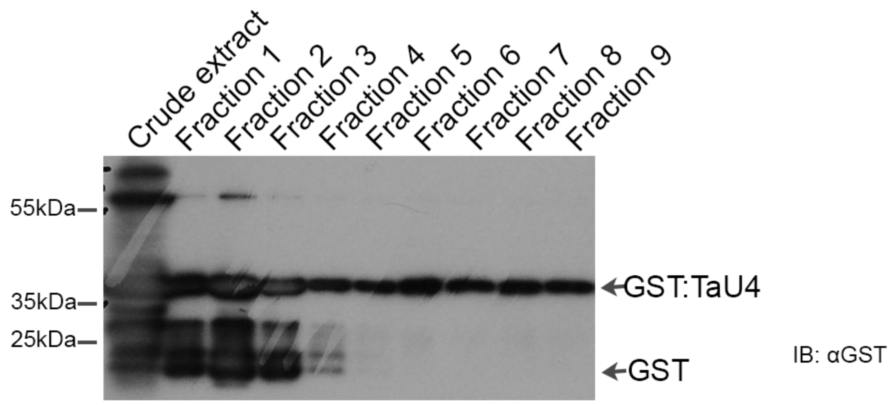

Supplemental figure 4. Purified fractions of GST:TaU4 analysed by western blotting. Crude extract from IPTG induced cell culture was purified using a GSTrap4B column. The crude extract and 9 eluted fractions are shown. Fractions 5-9 were used for the ubiquitin-charging assay as they did not contain any GST breakdown product. GST:TaU4 is 41kDa and GST is 26kDa. An anti:GST antibody was used to visualise the GST:TaU4.

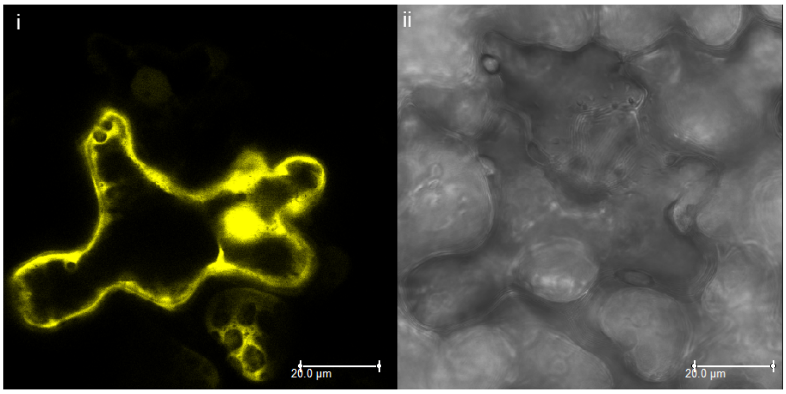

Supplemental figure 5. TaU4-YFP localises to the cytosol and nucleus in *N. benthamiana* leaf cells. As with figure 4, TaU4-YFP (pEARYLEYGATE104) was transiently expressed in *N. benthanmiana* leaves and visualised using confocal laser scanning microscopy **i** YFP fluorescence detected at 560-700nm. **ii** Brightfield image.

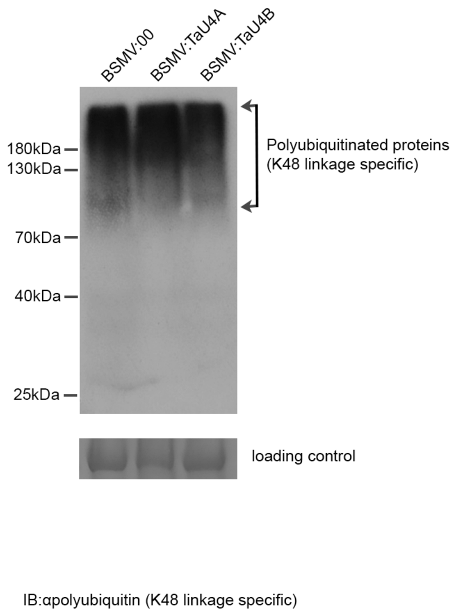

Supplemental figure 6. Total protein extract from TaU4 silenced wheat probed with a polyubiquitin K48 linkage-specific antibody. Total protein was extracted from *BSMV:00*, *BSMV:TaU4A* and *BSMV:TaU4B* silenced wheat leaves (3 weeks post silencing treatment). 30μg of each was loaded onto a gradient gel before western blotting with an antibody against specific K48 polyubiquitin chain (Abcam ab140601)(1:1000). Ponceau staining was used to ascertain equal loading of total protein extract.

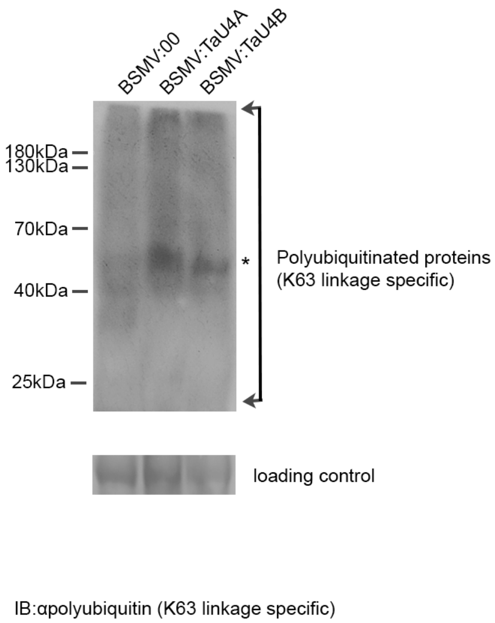

Supplemental figure 7. Total protein extract from *TaU4* silenced wheat probed with a polyubiquitin K63 linkage specific antibody. Total protein was extracted from *BSMV:00*, *BSMV:TaU4A* and *BSMV:TaU4B* silenced wheat leaves (3 weeks post silencing treatment). 30μg of each was loaded onto a gradient gel before western blotting with an antibody against specific K63-linkage specific polyubiquitin chains (Enzolifesciences HWA4C4)(1:1000). * Indicates accumulation of a protein band.

Ponceau staining was used to ascertain equal loading of total protein extract.

Primer	Primer sequence	Size amplicon
RT_18S F	CGGCTACCACATCCAAGGAA	200
RT_18S R	GCTGGAATTACCGCGGCT	
TaU4 F	ATGTCGACTCCTTCAAGGAAGAG	459
TaU4 R	TTAGTCTGCCGTCCAGCTCTGC	
TaU4A F	AAGGAAGTTTAACGCCCAGGCCGACCTAAAGCC	200
TaU4A R	AACCACCACCACCGTTGCGGCGCCCCGCTTATGCC	
TaU4B F	AAGGAAGTTTAATTTCTCGGATGTTTCACCCTAAC	200
TaU4B R	AACCACCACCACCGTGCGGTTGTACTCTCGCTTGTTC	
RT_TaU4A F	GGAGGCACGTTTAAGCTGAC	70
RT_TaU4A R	TAAACCGAACTGTTGGTGGC	
RT_TaU4B F	CGCTGCTGTGTGATCCTAAC	93
RT_TaU4B R	GCACTTTGCGGTTGTACTCT	

Supplemental table 1. Primer sequences used for cloning and qRT-PCR. qRT-PCR primers are indicated by the prefix RT.
